# Quality of vision and outcomes after bilateral implantation of pseudo-non diffracting beam IOL

**DOI:** 10.3389/fmed.2023.1085280

**Published:** 2023-03-06

**Authors:** Emilio Pedrotti, Erika Bonacci, Raphael Kilian, Camilla Pagnacco, Marco Anastasi, Mariacarmela Ventura, Giorgio Marchini

**Affiliations:** Ophthalmic Unit, Department of Neurosciences, Biomedicine and Movement Sciences, University of Verona, Verona, Italy

**Keywords:** cataract, spectacle independence, near vision, premium IOLs, extended depth of focus IOL

## Abstract

**Purpose:**

To analyze the objective and subjective visual performances of a new hybrid refractive/aspheric extended depth of focus (EDOF) intraocular lens (IOL).

**Methods:**

In this monocentric prospective study patients with bilateral cataracts underwent cataract surgery and were implanted with a Lucidis IOL (SAV-IOL SA, Neuchâtel, Switzerland) in both eyes, 1 week apart from each other. At 3 months from implantation postoperative evaluations included monocular and binocular uncorrected and distance-corrected distant (4 m), intermediate (80 cm, 67 cm) and near (40 cm) visual acuities (UDVA/DCVA, UI80-67VA/DCI80-67VA, UNVA/DCNVA), binocular defocus curves, halometry, contrast sensitivity and objective quality-of-vision measurements. Also, patients were also asked to complete the national eye institute refractive error quality of life (NEI-RQL-42) questionnaire.

**Results:**

Twenty-five patients (50 eyes) were included. The mean postoperative binocular UDVA, UI80VA, UI67VA and UNVA were-0.02 ± 0.13, 0.05 ± 0.09, 0.05 ± 0.08 and 0.03 ± 0.1 LogMar, and did not significantly differ from their corrected counterparts. On binocular defocus curves a VA ≥0.05LogMar was found between +0.50 and − 2.50 D of vergence, whereas the mean distance from the central stimulus on halometry was 1.23 ± 0.01. Mean ocular and corneal radical mean square at 4 mm were 0.31 ± 0.28 and 0.19 ± 0.07, respectively; whereas the mean Strehl ratio was 0.2 ± 0.09.

**Conclusion:**

Lucidis IOLs demonstrated excellent visual performances, especially at close distances while maintain good quality of vision, contrast sensitivity, and overall patient-satisfaction.

## 1. Introduction

In the last decades premium multifocal intraocular lenses (MFIOL) have been designed to meet the patients’ need for spectacle independence, however, these lenses frequently led to a bad quality of vision, especially at near and/or far distances. Other issues that have emerged through the years with these lenses consisted in the decrease of both contrast sensitivity and night vision, as well as in the frequent manifestation of visual phenomena such as halos, glare and starburst (especially with diffractive MFIOLs) (1–4). Recently, the need to overcome these concerns has led to the development of new technologies able to generate a single focal point with an extended depth of focus (EDOF). While improving far- and intermediate-distance spectacle independence, EDOF-IOLs are also said to be able to induce fewer visual phenomena (5, 6). However, these lenses are also known for the need of a small amount of positive spectacle correction at close distances (7).

The Lucidis IOL (Swiss Advanced Vision, SAV-IOL SA, Neuchâtel, Switzerland) is a new special hybrid refractive/aspheric EDOF IOL that has been created to overcome the limitation of near vision. However, until now only few studies have analyzed the outcomes of this lens and none of these has examined neither the defocus curve, nor the objective visual quality (8–10). The aim of this study was to examine the visual performances of the Lucidis IOL focusing on near vision, defocus curves, subjective and objective quality of vision and on the patient’s satisfaction 3 months after the surgery.

## 2. Patients and methods

This prospective interventional monocentric study adhered to the tenets of the Declaration of Helsinki and was approved by the local Ethics Committee (protocol 54,139). A written informed consent was obtained from all participating subjects after thorough explanation of the benefits and the risks related to the implantation of the IOL in study.

Inclusion criteria were the presence of significant bilateral cataracts, defined by a preoperative corrected distance visual acuity (DCVA) of 0.20 logMAR (20/32 Snellen) or worse, availability to undergo both surgeries 1 week apart from each other, an axial length between 22 and 23 mm and a preoperative regular corneal astigmatism of less than 1.00 diopter (D). We excluded patients younger than 18, those with any other concomitant or previous ocular disease, irregular astigmatism and those who had undergone previous ocular surgeries. Patients that had experienced intraoperative complications were excluded from the final analysis.

## 3. Clinical protocol

All patients underwent a thorough ophthalmological examination before surgery and 3 months after IOL implantation. The preoperative evaluation included measurement of monocular and binocular uncorrected and distance-corrected distant and near visual acuity (UDVA/DCVA at 4 m and UNVA/DCNVA at 40 cm, respectively) using the CSO Vision Charts V14.0 (CSO, Florence, Italy), measurement of the subjective refractive error, corneal tomography (MS-39, CSO, Firenze, Italy), optical biometry (Lenstar 900; Haag-Streit Diagnostics, Koeniz, Switzerland), Goldmann applanation tonometry, slit-lamp anterior segment examination, fundus examination under dilation and optical coherence tomography at the retinal plane (Spectralis OCT Heidelberg Engineering Inc., Heidelberg, Germany). Biometric values were used as inputs in the Kane formula to calculate the lens power, which in turn was selected targeting emmetropia (11).

Besides the binocular and monocular UDVA and DCVA at 4 m and the UNVA and DCNVA at 40 cm, the 3 months-postoperative visit, also included the uncorrected and best distance corrected intermediate visual acuity at 80 cm and 67 cm (UI80VA, DCI80VA, UI67VA and DCI67VA), binocular defocus curves, contrast sensitivity (CS) testing under photopic (80 cd/m2), mesopic (6 cd/m2), and scotopic (3 cd/m2) light conditions (CSV 1000 HGT; Vector Vision, Greenville, OH), ocular optical quality analysis by Pyramidal WaveFront-based sensor aberrometer (Osiris T Aberrometer, CSO, Firenze, Italy) and the halo test (Aston Halometer). After a slit lamp examination (to exclude the presence of posterior capsular opacity - PCO), patients were also asked to complete the National Eye Institute Refractive Error Quality of Life Instrument 42 (NEI-RQL-42) questionnaire.

Binocular defocus curves were obtained between +1.50 to −3.50 D using regular shifts of 0.50 D with respect to the 4 m DCVA and recording the best visual acuity for each step. To avoid memory effects, presenting letter sequences were randomized and patients’ eyes were occluded between each lens presentation (12). To analyze the ocular optical quality we used the Osiris T Aberrometer studying the ocular Root Means Square (RMS) and the Point-Spread-Function Strehl ratio (PSF Strehl ratio), which is defined as the ratio between the peak image intensity of the patient’s eye and that of an ideal eye (i.e., maximal intensity), limited only by diffraction (13). On the other hand, the purpose of the halo test is to measure in degrees how much a glaring source of light clouds a target. The halometer consisted of a light source (LED, Golden Dragon Pluc LCW W5AM.PC, 5000 K color temperature; Osram Licht AG, Munich, Germany) located in the center of an iPad4 tablet on which 0.3 logMAR (Snellen 20/40) letters were presented and moved toward the light source in 0.05-degree steps (14). To identify the halo area, patients stayed at 2 meters from the halometer in a dark room and were asked to recognize in succession the letters in six directions of orientation and separated by 60°. The cut-off value was collected for each direction. On slit lamp examination, if a grade 3 or higher PCO (According to Congdon’s study), (15) was detected, this was treated by YAG-laser capsulotomy and the 3 months evaluation was postponed 10 days thereafter. Finally, patients completed the NEI RQL-42 questionnaire to evaluate their quality of life in relation to their refractive error correction and visual acuity recovery (16). The questionnaire consists of 13 subscales with 42 items in 16 different question/response category formats.

### 3.1. Surgery

All cataract surgeries were performed by the same surgeon (E.P.) under topical anesthesia. A 2.2 mm corneal tunnel was created on the steepest meridian and was followed by a standard phaco-chop technique-surgery using the Stellaris phaco-platform (Bausch & Lomb Inc., Rochester, NY). The 12.4 mm Lucidis IOL was then placed in the capsular bag. The second surgical procedure was performed within 7 days from the first one. Prophylaxis consisted of an antibiotic and a nonsteroidal anti-inflammatory eye drop whereas the postoperative therapy also included topical steroid drops.

### 3.2. IOL

**Figure 1:**
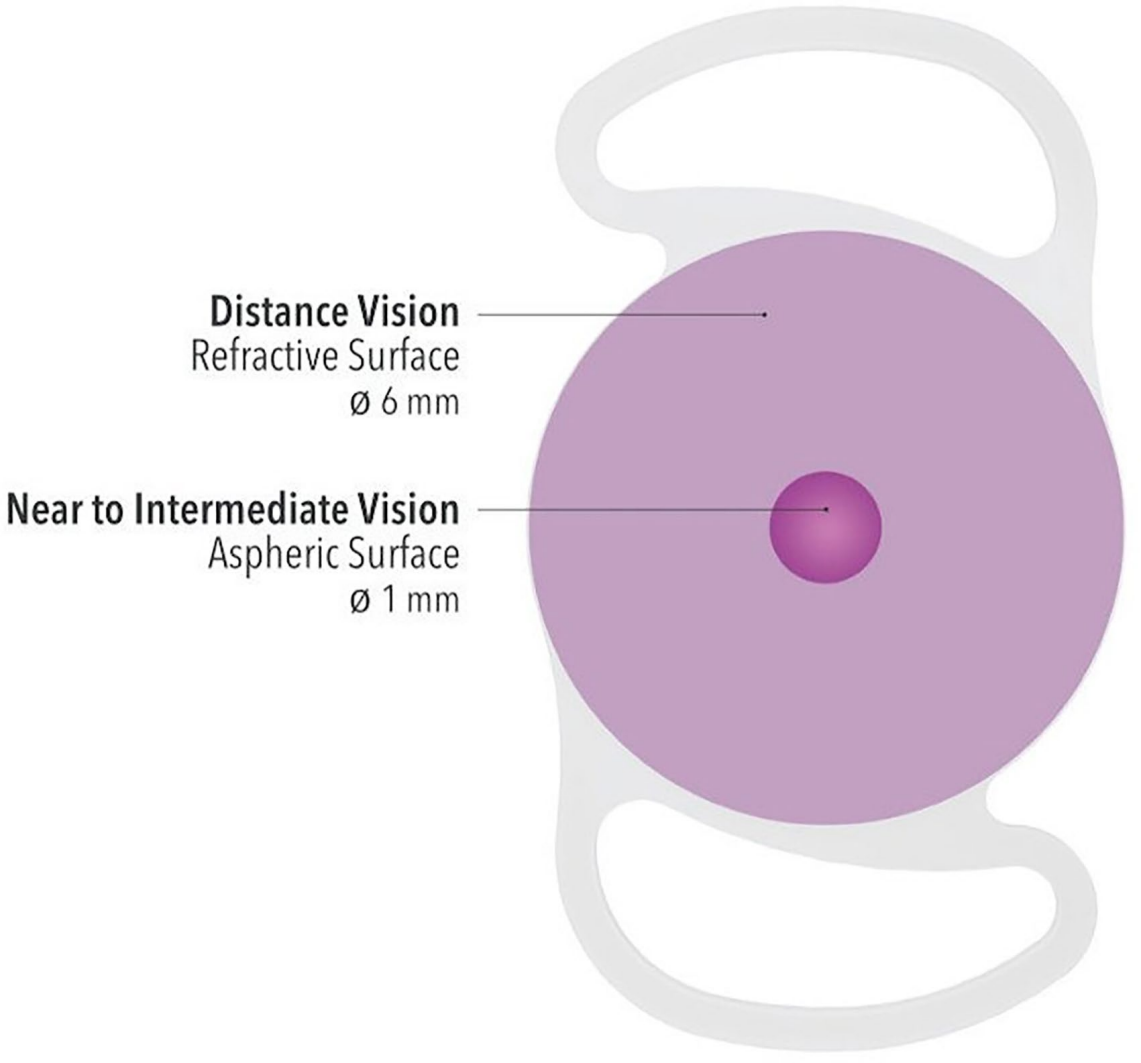
Schematic graph of the optic technology of Lucidis IOL with a central aspheric surface for near to intermediate vision and a larger refractive surface for distance vision.

The Lucidis lens (Swiss Advanced Vision, SAV-IOL SA, Neuchâtel, Switzerland)is a single-piece foldable hydrophilic acrylic lens with an optical diameter of 6.0 mm and a total diameter of 10.8 mm or 12.4 mm. The IOL has square edges with closed loop haptics and is designed to be implanted in the capsular bag. Its hybrid refractive/aspheric design, where a 1-mm aspheric central zone is surrounded by a 6-mm refractive ring (Figure 1), allows for a + 3.0 D addition power on top of the normal distance power, which ranges from +5.0 D to +30.0 D. In this study only the 12.4 mm-IOL was implanted in order to avoid IOL decentration.

### 3.3. Statistical analysis

Statistical analysis was performed using the IBM SPSS software version 24 for MacIntosh (IBM-SPSS). The Shapiro–Wilk test was used to determine data distribution. All quantitative results are reported as mean ± standard deviation for parametric distribution and as median ± interquartile range for non-parametric distribution. The t test for parametric distribution and the Mann–Whitney test for non-parametric distribution were used to compare the data. A *p* value lower than 0.05 was considered statistically significant.

The sample size was calculated based on monocular and binocular DCNVAs obtained from previous studies. With an estimated standard deviation of 0.13, a sample size of 50 patients produces a 95% confidence interval in DCNVA of 0.037. When the estimated standard deviation is 0.14, a sample size of 25 gives a 95% confidence interval of 0.06 (17). Postoperative data are presented at 3 months from implantation.

## 4. Results

Twenty-five patients (50 eyes) with a mean age (±SD) of 68 ± 10 years were included. Thirty-six percent of patients were male and 64% were female. The average spherical dioptric power of the implanted IOLs was 19.01 ± 4.29 D (median: 19.0 D, range: 12.5 to 26.5 D).

There were no major postoperative or intraoperative complications.

At 3-months from implantation, a grade 3 PCO was found in 1 eye (2.1%) and a YAG-laser capsulotomy was performed.

### 4.1. Visual outcomes

The mean postoperative subjective refractive spherical equivalent was −0.36 ± 0.39 D and laid within ±0.51 D in 58% of eyes and within ±1.00 D in 100% of cases.

Table 1 summarizes both the monocular and binocular uncorrected and distance corrected VA-results.

**Table 1:** Postoperative monocular and binocular visual acuities.

	Monocular VA	*p*	% of patients reaching a VA > 20/40	% of patients reaching a VA > 20/25
UDVA	0.04 ± 0.13	0.17	93	54
DCVA	−0.04 ± 0.08		100	89
UI80VA	0.07 ± 0.09	0.53	98	46
DCI80VA	0.09 ± 0.09		98	37
UI67VA	0.08 ± 0.11	0.44	87	41
DCI67VA	0.11 ± 0.11		83	43
UNVA	0.07 ± 0.12	0.82	91	41
DCNVA	0.07 ± 0.11		89	46
	Binocular VA	*p*	% of patients reaching a VA > 20/40	% of patients reaching a VA > 20/25
UDVA	−0.02 ± 0.13	0.87	100	70
DCVA	−0.07 ± 0.09		100	87
UI80VA	0.05 ± 0.09	0.75	96	52
DCI80VA	0.06 ± 0.07		100	42
UI67VA	0.05 ± 0.08	0.89	96	52
DCI67VA	0.04 ± 0.09		96	57
UNVA	0.03 ± 0.1	0.99	100	65
DCNVA	0.00 ± 0.08		100	74

*p* values show no statistical differences between distance corrected and uncorrected visual acuities.

The differences between the mean binocular and monocular UDVA and UI80VA, UI67VA and UNVA were not statistically significant (*p* = 0.26, *p* = 0.24 and *p* = 0.24 and *p* = 0.31, *p* = 0.83 and *p* = 0.84, respectively).

### 4.2. Defocus curve

**Figure 2:**
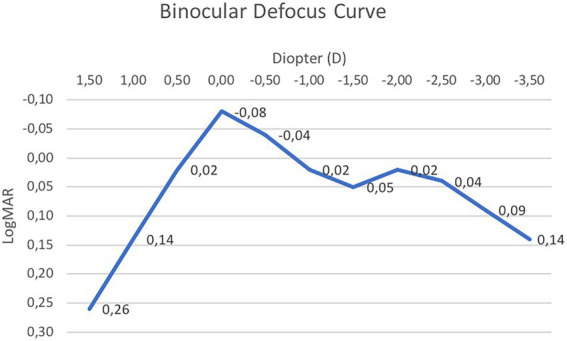
Mean binocular defocus curve.

Figure 2 shows the mean binocular defocus curve at 3 months after surgery. Visual acuity was found to be higher than or equal to 0.05 logMar between +0.50 and − 2.50 D of vergence, showing the deepest point at −1.50 D. However, neither the difference in VA between 0.00 and − 1.5 D, nor that between −1.5 and − 2 D, were statistically significant (*p* = 0.08 and *p* = 0.11, respectively).

### 4.3. Contrast sensitivity outcomes

**Figure 3:**
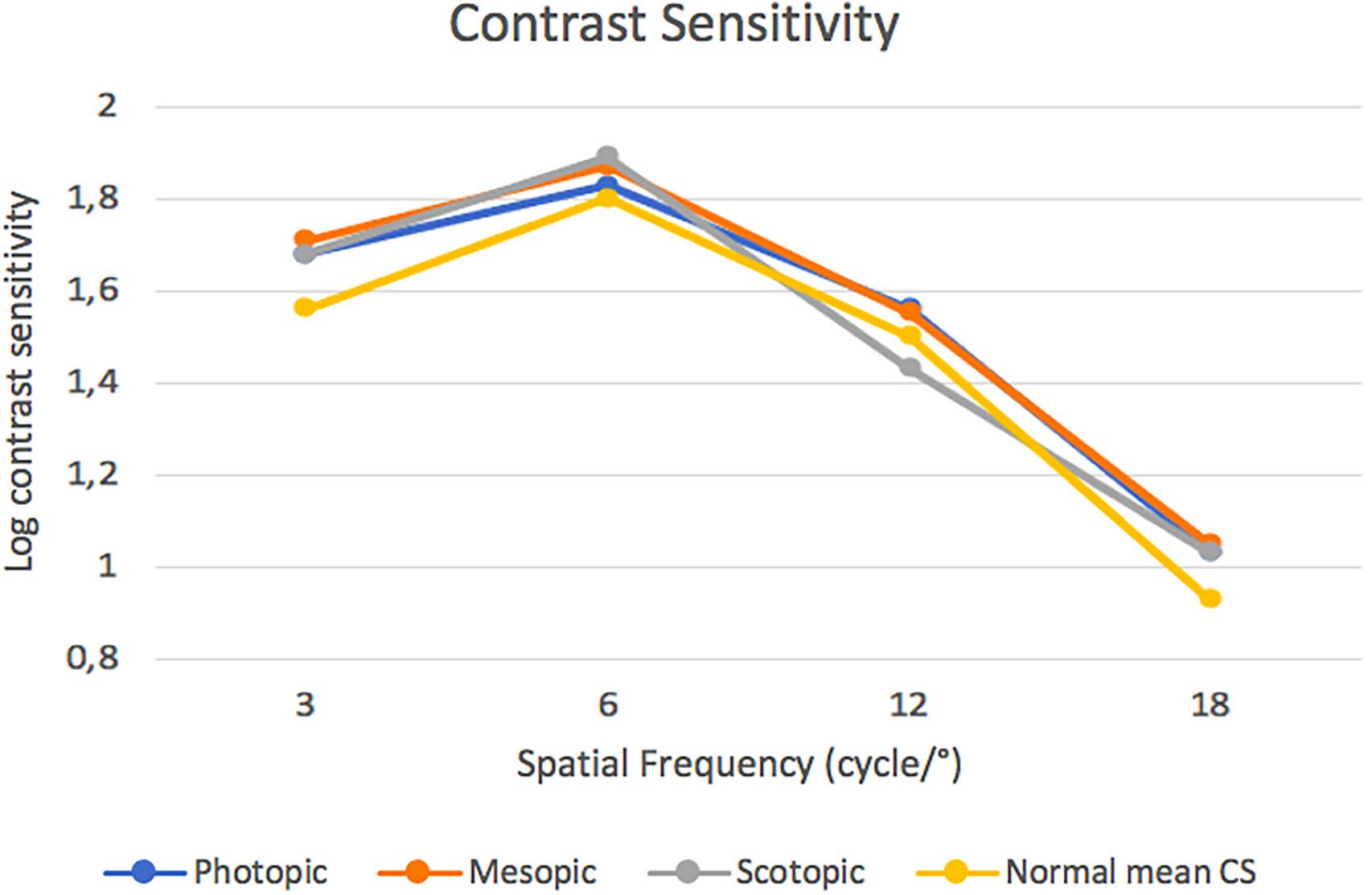
Postoperative contrast sensitivity function measured under scotopic, mesopic and photopic conditions.

Figure 3 presents the binocular CS function measured under scotopic, mesopic, and photopic light conditions. There were no statistically significant differences among the three conditions at any of the studied spatial frequencies (e.g., in the scotopic vs. photopic condition at 12 cpg the *p* value was 0.26). Mean CS values of a population ranging from 50 to 75 years of age were also taken into account and the performance of this IOL was statistically significant better at 3 cpd in photopic, mesopic and scotopic condition *p* = 0.01, *p* = 0.004 and *p* = 0.03, respectively (18).

### 4.4. Halometry

**Figure 4:**
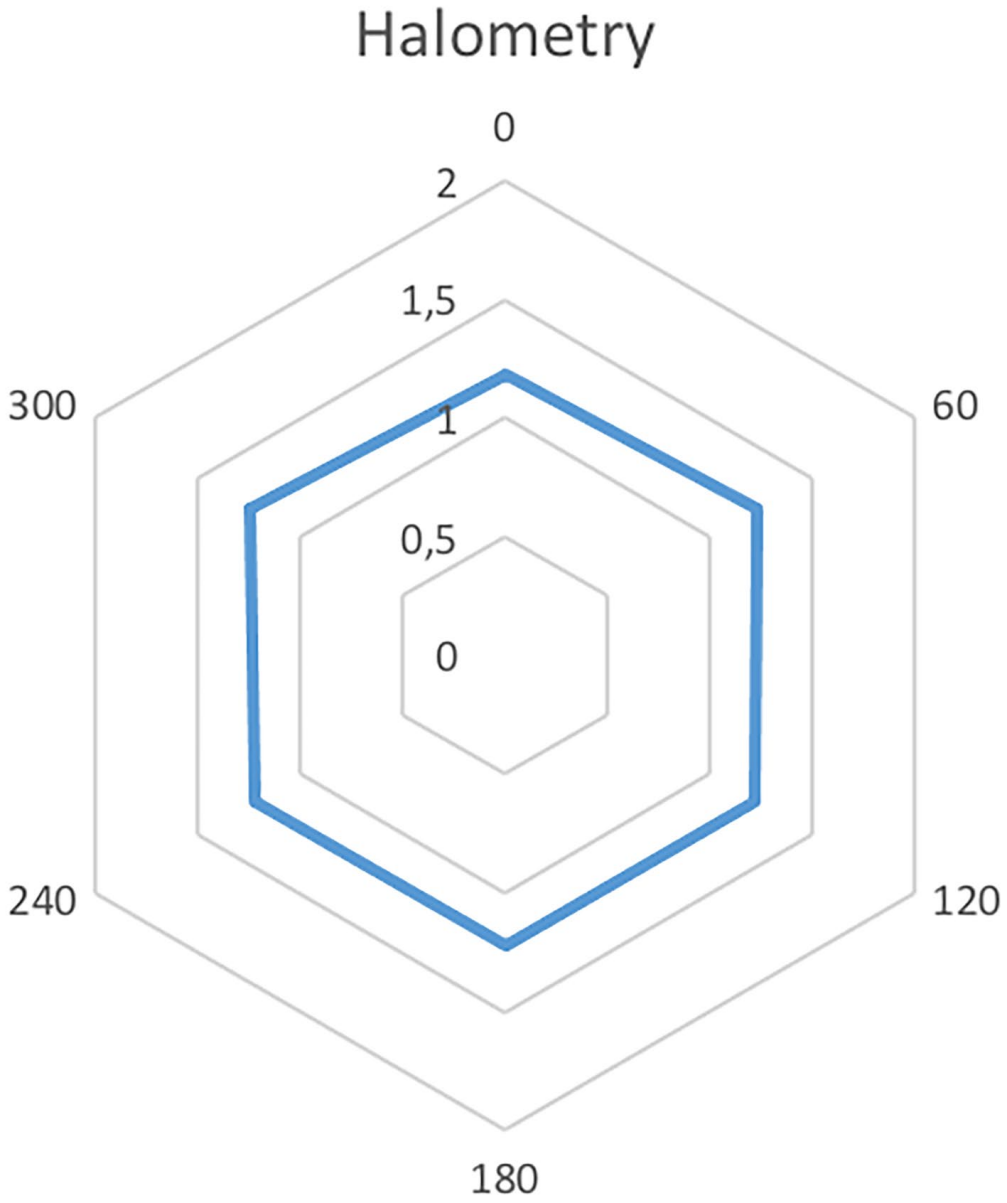
Six-vertex mean halometry for the hybrid refractive/aspheric Lucidis IOL.

The mean distance from the central stimulus was 1.23 ± 0.01. Figure 4 presents the mean halometric cut-off values for each of the six axes.

### 4.5. Quality of vision parameters

At 3 months from the operation the mean ocular and corneal RMS at 4 mm were 0.31 ± 0.28 (range: 0.09–1.5) and 0.19 ± 0.07 (range: 0.07–0.5), respectively; whereas the mean PSF Strehl ratio was 0.2 ± 0.09 (range: 0.03–0.41).

### 4.6. Quality of life outcomes

The NEI RQL-42 evidenced high subjective satisfaction results for all the items, especially for suboptimal correction, activity limitations, glare, appearance, far vision, dependence on correction and satisfaction with correction (Table 2).

**Table 2:** Postoperative QoL scores on the 13 subscales of the NEI-RQl-42.

**Parameter**	
Clarity of vision	
Mean ± SD	78.50 ± 26.97
Median (range)	100 (0.00 to 100.00)
Expectations	
Mean ± SD	52.94 ± 44.28
Median (range)	50.00 (0.00 to 100.00)
Near vision	
Mean ± SD	78.57 ± 24.19
Median (range)	75.00 (0.00 to 100.00)
Far vision	
Mean ± SD	81.74 ± 25.75
Median (range)	100.00 (0.00 to 100.00)
Diurnal fluctuations	
Mean ± SD	76.61 ± 28.09
Median (range)	87.50 (0.00 to 100.00)
Activity limitations	
Mean ± SD	91.67 ± 23.36
Median (range)	100.00 (0.00 to 100.00)
Glare	
Mean ± SD	86.76 ± 21.86
Median (range)	100.00 (50.00 to 100.00)
Symptoms	
Mean ± SD	70.15 ± 28.51
Median (range)	75.00 (0.00 to 100.00)
Dependence on correction	
Mean ± SD	72.38 ± 36.21
Median (range)	100.00 (0.00 to 100.00)
Worry	
Mean ± SD	52.18 ± 35.40
Median (range)	50.00 (0.00 to 100.00)
Suboptimal correction	
Mean ± SD	93.75 ± 13.86
Median (range)	100.00 (50.00 to 100.00)
Appearance	
Mean ± SD	84.19 ± 30.15
Median (range)	100.00 (0.00 to 100.00)
Satisfaction with correction	
Mean ± SD	78.89 ± 18.75
Median (range)	80.00 (40.00 to 100.00)

## 5. Discussion

Extended depth of focus technology is among the most effective proposed methods to enhance spectacle independence after cataract surgery. Nevertheless, when it comes to near vision, these lenses are usually outperformed by MFIOLs, which, however, are often burdened by annoying light phenomena (1, 2).

In this study, the Lucidis IOL has shown to be able to strengthen the near-distance VA at the expense of a slight decrease in the intermediate vision. Indeed, 74, 57 and 42% of patients reached a binocular VA higher than 20/25 at 40, 67 and 80 cm, respectively. This result was confirmed by the trend of the defocus curve and seems to be in accordance with the current literature (4–6). Authors would like to underline that these results appear to be in agreement with the available literature on Lucidis IOLs, as to our knowledge currently no study has ever reported the DCIVA, but only the UIVA (without specifying how many cm it was run) and none performed defocus curves.

Although a direct comparison was not performed, when considering the results of other EDOF IOLs, it is striking how these are usually characterized by a regular downslope in the myopic portion of the defocus curve, reaching the lowest performances around −2.50 D; indeed, patients often need a spherical addition of 1 D in order to achieve the optimal near-distance VA (7, 19, 20). Meanwhile, in our study, at −2.50D of vergence, the defocus curve showed a mean VA of little less than 0.05 LogMar. With regards to intermediate VAs, on the other hand, our results do not significantly differ with those of other EDOF IOLs.

When considering an extended range of vision (ERV) IOL (21) such as the TECNIS Symphony, it seems like Lucidis IOLs perform better at far and near distances, whereas the former performs better at intermediate distances (22, 23).

Surprisingly, the Lucidis IOL showed comparable performances to the tri-quadrifocal Enlighten Panoptix IOL at the 40 cm distance (0.14 ± 0.04 and 0.00 ± 0.08 for Enlighten and hybrid IOLs, respectively) and performed even better than this IOL at intermediate distances (0.10 ± 0.03 and 0.04 ± 0.09 for Enlighten and Lucidis IOL, respectively) (22).

This outstanding performance is probably related to the special hybrid design of these lenses. The main optical propriety is due to the central aspheric portion of this IOL which is able to create a peak of light *via* constructive light wave interference, whereas the periphery maintains a refractive surface. The lens therefore acts as an axicon (Bessel like ray of light). The system altogether results in the formation of a pseudo non diffracting beam which starts to diverge after some distance from the lens itself, therefore covering the whole range of vision (i.e., from near-intermediate to far distances). An axicon lens is an optical element first introduced in 1954 by McLeod, (24) able to transform a laser beam into a ring-shaped distribution, resulting in a beam of focal fields that allow a continuous vision from intermediate to short distances.

Interestingly, these visual performances are achieved while preserving a good quality of vision. Indeed, the ocular RMS was 0.31 ± 0.28, with corneal component of 0.19 ± 0.07 and a mean internal RMS of 0.12 (i.e., ocular RMS – corneal RMS). The RMS is closely related to Zernike polynomials and its minimum value is 0, which represents the ideal wavefront condition. Even though the measurement was taken using different instruments, the internal RMS of the Lucidis IOL results to be lower than both the ZXR00s-TECNIS Symphony’s (0.15 ± 0.06) and the tri-quadrifocal Enlighten Panoptix’s (0.18 ± 0.06), measured in a previous study of ours (22). The RMS results of the current study differ from those found by Rabinovich et al. (10) on Lucidis IOLs. However, the latter study has several limitations, such as its retrospective design and the absence of a precise description of what RMS evaluation had been carried out and what instrumentation was used, so a reliable comparison between our results is not feasible. Nonetheless, the total RMS found in this study (i.e., 0.18 ± 0.1) seems to be better than that obtained with aberrometric EDOFs, multifocal diffractive and refractive IOLs (13).

Lucidis IOLs however, showed worse performances than the aberrometric EDOF Mini Well IOL (SIFI S.p.A., Catania, Italy) and the Enlighten IOL in terms of mean PSF Strehl ratio and CS at the lower spatial frequencies (3, 22).

In addition, halometry results show that the Lucidis IOL performs very similarly to monofocal IOLs for all mean cut-off values (17). Even though no direct comparison has been performed, the NEI RQL-42 questionnaire-results seem to show higher subjective satisfaction with the Lucidis IOL than with aberrometric EDOF, ERV and Enlighten IOLs with regards to “glare” evaluation. Despite patients reporting good levels of satisfaction after bilateral implantation of this hybrid lens, all other items in the questionnaire seem to show better results with aberrometric EDOF IOLs (17, 22).

Among the limitations of this study it is worth mentioning its limited number of patients and the absence of a direct comparison with the other type of IOLs.

To conclude, Lucidis IOLs demonstrated a good safety profile and excellent visual performances at all distances, but especially at near distances, while also allowing a good quality of vision.

## Data availability statement

The original contributions presented in the study are included in the article/Supplementary material, further inquiries can be directed to the corresponding author.

## Ethics statement

The studies involving human participants were reviewed and approved by Comitato etico per la Sperimentazione Clinica (CESC) delle Province di Verona e Rovigo. The patients/participants provided their written informed consent to participate in this study.

## Author contributions

All authors made substantial contributions to conception and design, acquisition of data, analysis, and interpretation of data. They all took part in drafting the article or revising it critically for important intellectual content and agreed to submit it to the current journal. All authors gave final approval of the version to be published and agreed to be accountable for all aspects of the work.

## Conflict of interest

The authors declare that the research was conducted in the absence of any commercial or financial relationships that could be construed as a potential conflict of interest.

## Publisher’s note

All claims expressed in this article are solely those of the authors and do not necessarily represent those of their affiliated organizations, or those of the publisher, the editors and the reviewers. Any product that may be evaluated in this article, or claim that may be made by its manufacturer, is not guaranteed or endorsed by the publisher.
